# Hospital Meetings

**Published:** 1903-11-21

**Authors:** 


### HOSPITAL MEETINGS.

### EVELINA HOSPITAL FOR SICK CHILDREN.

A SPECIAL court of the Governors of this hospital was held on Wednesday, November 4th, in order to confirm the new constitution and rules passed at the last court. The chair was taken by Mr. Charles Wightman, chairman of the committee of management, and the motion confirming the alterations made in the constitution and rules of the hospital having been put to the meeting and carried unanimously, the proceedings terminated.

### BRITISH HOME AND HOSPITAL FOR INCURABLES.

THE half-yearly meeting of the governors of this institution was held on the 12th inst., Mr. J. Hampton Hale, treasurer and deputy-chairman, presiding.

The Chairman stated that the efficiency of the Home had been kept well up to date, but there were still a few things needed to improve the appearance and usefulness of the Home and to increase the comfort of the patients, and one of those was the installation of electric light throughout the building. This, through the generosity of a lady subscriber, who had given a donation of £300, they were enabled to take in hand at once, but they would need a further sum of £100 to complete the undertaking. With reference to the visit of the Queen to the Home to receive her coronation gift, they had been disappointed that her Majesty had been unable to undertake this visit during the past year on account of her absence from England. They had, however, received an intimation from the Queen's private secretary, Mr. Greville, that her Majesty hoped to visit the Home early next year. Her Majesty had asked the board to allow her, in anticipation of her visit, to exercise her privilege of nominating an inmate to the Home. This request the board had acceded to. After alluding to the regret the board felt at the resignation of Dr. Rugg, the medical officer, who had been connected with the Home for over 24 years, the Chairman proceeded to touch upon their financial position. They were now £500 in debt to their bankers, and unless help was forthcoming they would be in a worse condition by the New Year. They required a sum of £50 a day to carry on the Home, £580 a month going in pensions. A list of those elected to fill vacancies in the Home having been read, the proceedings closed, after a vote of thanks to the Chairman had been passed.

